# Beyond the trauma: a scoping review of nursing interventions for improving psychological well-being in adolescents bullying victims

**DOI:** 10.1186/s12912-025-03642-4

**Published:** 2025-07-25

**Authors:** Erviana Zefanya Agatha, Iyus Yosep, Taty Hernawaty, Rohman Hikmat, Thomas Ferdinanto, Riska Nur Assyifa Febrianti, Muchamat Irawan Danisholehudin

**Affiliations:** 1 Bachelor Program of Nursing, Faculty of Nursing, Universitas Padjadjaran, Sumedang, Jawa Barat Indonesia; 2 Department of Mental Health, Faculty of Nursing, Universitas Padjadjaran, Sumedang, Jawa Barat Indonesia; 3 Nursing Department, Faculty of Health Science, Universitas ‘Aisyiyah Bandung, Bandung, Jawa Barat Indonesia; 4 Faculty of Nursing, Universitas Padjadjaran, Jl. Raya Ir. Soekarno KM. 21, Hegarmanah, Jatinangor, Sumedang, West Java 45363 Indonesia

**Keywords:** Psychological well-being, Bullying, Nursing interventions, Adolescents

## Abstract

**Background:**

Bullying is a critical issue that deeply affects adolescents’ psychological well-being, leading to both immediate and long-term emotional and physical harm. Effective nursing interventions are essential for mitigating these impacts and improving mental health outcomes for victims. By integrating comprehensive, evidence-based approaches, nurses can play a pivotal role in alleviating the adverse effects of bullying, supporting adolescents through their recovery, and fostering a more resilient and healthier future.

**Purpose:**

This scoping review aims to explore nursing interventions for improving psychological well-being in adolescents bullying victims.

**Methods:**

Adhering to the PRISMA Extension for Scoping Review (PRISMA-ScR) guidelines, we conducted a comprehensive scoping review. Literature was sourced from major databases, including PubMed, CINAHL (via EBSCOhost), ScienceDirect, and Scopus, using targeted keywords related to bullying and psychological well-being. The review focused on studies involving adolescents aged 10–20 from diverse international contexts.

**Results:**

Out of 28092 articles screened, 11 articles met the inclusion criteria and were thoroughly analyzed. These studies involved a broad range of participants, including students, parents, and teachers, with sample sizes varying from 66 to 39793. The review highlights a range of nursing interventions that combine creative approaches, self-development techniques, and social support, demonstrating significant positive effects on adolescents’ psychological well-being.

**Conclusion:**

This scoping review underscores the potential of multifaceted nursing interventions to mitigate the effects of bullying and enhance the psychological well-being of adolescent victims. Tailoring approaches to individual needs and contexts proves essential for achieving the most impactful results. By integrating creative, self-developmental, and supportive strategies, nursing interventions can provide a robust framework for addressing bullying and promoting mental health in adolescents.

## Introduction

Bullying is defined as unwanted aggressive behavior by another youth or group of youths, involving an observed or perceived power imbalance and repeated multiple times or highly likely to be repeated [1]. It can result in physical, psychological, social, or educational harm to the victim. Bullying in adolescence is a health issue that can increase the risk of health problems, negative social outcomes, and low educational achievement, both in childhood and adolescence, and can take the form of physical contact, words, or more subtle actions [1, 2]. The number of reported bullying cases continues to rise each year. Although not all cases are documented, existing data indicate that bullying is a widespread problem. According to a 2023 global study by UNESCO, one in three students worldwide experiences bullying at school each month [3]. Among adolescents aged 13–15 years, over 36% have been involved in physical fights with peers, and nearly one in three students has been physically attacked at least once a year. A survey conducted by UNICEF in 2020 in Indonesia, involving 2,777 young people aged 14–24, found that 45% reported having experienced cyberbullying [4]. One of the countries with the highest bullying rates in Europe is the United States. According to a 2024 report from the CDC (Centers for Disease Control and Prevention), nearly 14% of public schools reported that bullying is a disciplinary issue occurring daily or at least once a week. Bullying incidents are most frequently reported in middle schools (28%), followed by high schools (16%), combined schools (12%), and elementary schools (9%) (CDC, 2024) [5].

Bullying in schools can be influenced by various interconnected factors. Recent research indicates that an unstable or unsupportive family environment can increase the risk of bullying, both as a perpetrator and as a victim. Children who experience violence at home or lack emotional attention from their parents are more vulnerable to bullying [6]. Additionally, individual factors such as low self-esteem, mental health issues, or certain physical conditions can increase the likelihood of someone becoming a target of bullying. Children who struggle with emotional regulation or feel helpless often become targets of bullying, while those who feel oppressed or marginalized may be more inclined to bully others as a form of compensation [7].

Bullying is often linked to psychological well-being due to its significant negative impact on the victim’s psychological state. Psychological well-being is a condition characterized by feelings of happiness, satisfaction, low levels of stress, physical and mental health, and a good quality of life [8]. Several studies show that children and adolescents who are both victims and perpetrators of cyberbullying represent a distinct group with the highest risk of psychosocial problems, such as symptoms of depression and anxiety, and lower levels of psychological well-being [9]. Cyberbullying is a form of bullying that occurs through digital platforms, such as social media, messaging apps, gaming platforms, or other online communication tools. Unlike traditional bullying, which typically involves face-to-face interactions, cyberbullying uses technology to harass, threaten, or humiliate individuals. It can include actions such as sending harmful messages, spreading rumors, or sharing private information without consent. The anonymity and broad reach of digital platforms can amplify the psychological harm caused by cyberbullying [10]. As with bullying in general, cyberbullying can also impact long-term mental health and reduce quality of life, demonstrating the importance of early intervention and support to address the adverse effects of these actions.

Psychological well-being plays a crucial role in mitigating the negative impact of bullying on victims. Recent studies highlight the importance of interventions aimed at enhancing the psychological well-being of those affected by bullying as both victims and perpetrators. For example, interventions focused on improving the psychological well-being of school violence victims have shown significant benefits, with cognitive-behavioral approaches proving effective in reducing symptoms such as depression and anxiety and enhancing overall life satisfaction [11]. Additionally, research indicates that enhancing psychological well-being helps victims manage emotional stress, form healthier relationships, and build resilience, which ultimately positively affects their mental health [12]. These findings are supported by studies revealing that psychological well-being is critical for both perpetrators and victims of bullying, as both groups experience a decline in well-being, albeit in different ways [13]. Overall, implementing targeted interventions to enhance psychological well-being can facilitate the recovery and resilience of victims of bullying, thereby improving their quality of life and mental health.

While many studies have examined the impact of bullying on psychological well-being, there remains a significant gap in the development of specific interventions by nurses to address this issue. Research indicates that although the effects of bullying on mental health are widely recognized, specific intervention approaches that focus on the psychological needs of victims are still rarely applied in nursing practice. The lack of specially designed interventions can exacerbate the long-term psychological impact of bullying, such as an increased risk of depression and anxiety [14]. Additionally, many existing programs tend to focus on general bullying prevention without giving adequate attention to personalized psychological support for victims [15]. This gap highlights the need for the development and implementation of more targeted, evidence-based intervention strategies to effectively enhance the psychological well-being of bullying victims.

As health workers, nurses have an important role in dealing with bullying behavior. Nurses can collaborate with various parties, government, teachers, health workers, parents, community environment, and the children themselves, to provide education on the prevention and treatment of trauma caused by *bullying* [16]. Through their capacity as educators and advocates, nurses contribute to increasing understanding and awareness of the importance of having good psychological well-being while providing support to victims [17]. In addition, nurses also act as counselors who focus on prevention and intervention efforts, with the aim of reducing the incidence of *bullying*, especially among children and adolescents [18].

Previous scoping review have shown that nursing interventions can prevent bullying behavior and overcome trauma in victims of violence [16]. Previous study have highlighted that nursing interventions can contribute to the mental health recovery of bullying victims by providing emotional support, psychoeducation, and resilience-building strategies [18]. For instance, cognitive-behavioral interventions led by nurses significantly reduced symptoms of anxiety and depression in adolescents who experienced bullying [13]. Similarly, previous study reported that school-based nursing programs incorporating mindfulness and stress management techniques improved emotional regulation and self-esteem among victims of peer aggression [11]. However, there are no reviews that explain how nursing interventions are carried out to comprehensively improve psychological well-being in victims of bullying. Psychological well-being is an important aspect that determines the long-term recovery and quality of life of victims [19]. Given the important role of nursing in providing holistic support that includes physical, emotional, and social aspects, a scoping review is needed to explore various nursing interventions that are effective in improving the psychological well-being of victims of bullying [18].

Through a holistic approach, nurses can work with teachers and parents to understand the factors that influence children’s mental health and risk of bullying. By identifying unmet needs, nurses can expand their knowledge of the psychological aspects that are crucial in supporting children’s mental well-being. This collaboration is expected to create a strong evidence base for further scoping reviews and research, to strengthen psychological support and build safe and bullying-free communities.

## Materials and methods

### Study design

This study follows the methodological framework for scoping reviews as outlined by Arksey and O’Malley, incorporating updates from Levac et al. and the Joanna Briggs Institute (JBI) guidelines. The review process consists of five core stages: (1) identifying the research question, (2) identifying relevant studies, (3) study selection, (4) data charting and mapping, and (5) collating, summarizing, and reporting the results. This structured approach ensures a comprehensive exploration of the available evidence on nursing interventions aimed at enhancing psychological well-being among adolescents affected by bullying. Additionally, the PRISMA Extension for Scoping Reviews (PRISMA-ScR) was used to guide the transparent and systematic reporting of the review process.

### Search methods

Review question: “What nursing interventions can be implemented to enhance psychological well-being among adolescent bullying victims?” Relevant literature for this study was obtained through an in-depth exploration of four major databases: PubMed, CINAHL (via EBSCOhost), ScienceDirect, and Scopus on April – May 2024. Searches were conducted using specific keywords that included “teenagers” OR “adolescents” OR “bullying victimization” “young adults” OR “teens” OR “youth” AND “interventions” OR “counseling” OR “social supports” OR “Psychological Well-Being” OR “Psychological health” OR “coping strategies” AND “bullying” OR “reduced bullying” OR “increased psychological well-being”. The primary research question guiding this study was: “What interventions can be implemented to enhance psychological well-being in adolescents bullying victims?” This question served as a framework for evaluating and filtering the literature related to this topic. The consultation with the research librarian contributed to refining the search strategy, particularly in selecting precise terms and ensuring compatibility across databases. This step was critical in enhancing the rigor of the systematic search and ensuring no significant studies were overlooked. Searching strategy in this study are:

- PubMed: (“Teenagers” OR “Adolescents” OR “Young adults” OR “Teens” OR “Youth” OR “School-age children” OR “Early adolescents”) AND (“Bullying victimization” OR “Peer bullying” OR “School bullying” OR “Cyberbullying” OR “Relational bullying”) AND (“Interventions” OR “Counseling programs” OR “Therapeutic interventions” OR “Group therapy” OR “Social support” OR “Peer support” OR “Behavioral programs”) AND (“Psychological well-being” OR “Mental health” OR “Emotional resilience” OR “Stress coping” OR “Self-esteem improvement”).
- CINAHL: (“Teenagers” OR “Adolescents” OR “Young adults” OR “Teens” OR “Youth” OR “School-age children” OR “Early adolescents”) AND (“Bullying victimization” OR “Peer bullying” OR “School bullying” OR “Cyberbullying” OR “Relational bullying”) AND (“Interventions” OR “Counseling programs” OR “Therapeutic interventions” OR “Group therapy” OR “Social support” OR “Peer support” OR “Behavioral programs”) AND (“Psychological well-being” OR “Mental health” OR “Emotional resilience” OR “Stress coping” OR “Self-esteem improvement”).
- ScienceDirect: TITLE-ABS-KEY((“Teenagers” OR “Adolescents” OR “Young adults” OR “Teens” OR “Youth” OR “School-aged children”) AND (“Bullying victimization” OR “Peer harassment” OR “Bullying behavior” OR “Cyberbullying”) AND (“Interventions” OR “Therapeutic programs” OR “Counseling” OR “Social engagement programs” OR “Psychosocial support”) AND (“Psychological well-being” OR “Emotional health” OR “Mental health” OR “Coping strategies” OR “Resilience”)).
- Scopus: ALL((“Teenagers” OR “Adolescents” OR “Young adults” OR “Teens” OR “Youth” OR “High school students”) AND (“Bullying victimization” OR “Peer victimization” OR “School bullying” OR “Cyberbullying”) AND (“Interventions” OR “Programs” OR “Counseling” OR “Social support” OR “Psychosocial support”) AND (“Psychological well-being” OR “Mental health” OR “Coping strategies” OR “Resilience”)).

### Inclusion and exclusion criteria

The PCC framework identified the population as adolescents, the concept as psychological well-being interventions, and the context as bullying-related situations. Articles included in this review were selected based on predefined inclusion and exclusion criteria to ensure relevance and methodological rigor. The inclusion criteria covered empirical studies with quantitative, qualitative, or mixed-methods designs that examined nursing interventions for improving psychological well-being in adolescent bullying victims. The population of interest was adolescents aged 10–20 years who had experienced physical, verbal, or cyberbullying. Studies were included if they reported outcomes such as improved psychological well-being, reduced depression or anxiety, or enhanced self-esteem. To ensure the inclusion of recent and relevant interventions, only studies published between 2019 and 2024 were considered. Additionally, articles were required to be published in English to ensure accessibility to internationally recognized research.

Exclusion criteria were applied to filter out studies with inadequate methodology, which was defined as those lacking a clear research design, insufficient sample sizes, absence of outcome measures, or poor methodological rigor, such as a lack of control groups in experimental studies or unclear intervention details. Studies were also excluded if they did not focus on bullying victims or psychological well-being interventions, were not available in full text, or were published before 2019.

The screening process was conducted in two phases. In the first phase, two independent reviewers screened the titles and abstracts of all retrieved articles using Mendeley for reference management. Articles that clearly did not meet the inclusion criteria were excluded at this stage. In the second phase, the full texts of potentially eligible articles were reviewed in detail to confirm their inclusion based on the predefined criteria. Any discrepancies between the reviewers were resolved through discussion, and if consensus could not be reached, a third reviewer was consulted. This systematic approach ensured the reliability and validity of the study selection process.

### Data extraction

The data extraction process for the scoping review was conducted by two authors, EZA and IY, who independently extracted the data to ensure objectivity and minimize potential bias. As experts in their field, their independent analyses contributed to the credibility and thoroughness of the review. When discrepancies or disagreements occurred during the data extraction process, the authors engaged in a discussion to reach a consensus. If consensus was still not achieved, a third author, TH, was invited to assist in resolving any outstanding issues.

The articles were manually extracted by EZA and IY using a pre-prepared data extraction table. Prior to entering data into the table, both authors performed a preliminary analysis and summarized the content of each article. The data extraction table was designed to capture essential details such as the author names, publication year, country of origin of the research, research design, population and sample characteristics, procedures used, type of intervention, and research outcomes. Once the information was entered into the table, the authors provided a detailed explanation of their analysis, including a description of the interventions and their impact on enhancing psychological well-being in bullying victims. This process ensured a comprehensive and accurate representation of the data relevant to the review’s objectives.

### Data analysis

After thoroughly reviewing the collected articles, all authors conducted an in-depth thematic analysis [21]. In this process, the authors identified, categorized, and synthesized various nursing interventions implemented to enhance psychological well-being in adolescent victims of bullying. Thematic analysis was carried out systematically by coding key themes from the extracted data. To ensure analytical rigor, the authors engaged in ongoing discussions to resolve discrepancies and refine the categorization of interventions. Following this process, the extracted data were synthesized, and interventions were classified into categories based on similarities in methods or approaches. Each category was described in detail to provide a clear understanding of the strategies used and their outcomes in addressing bullying-related psychological well-being issues. This approach ensures a structured and comprehensive overview of the effectiveness of various interventions applied in the context of adolescent bullying.

## Results

**Fig. 1:**
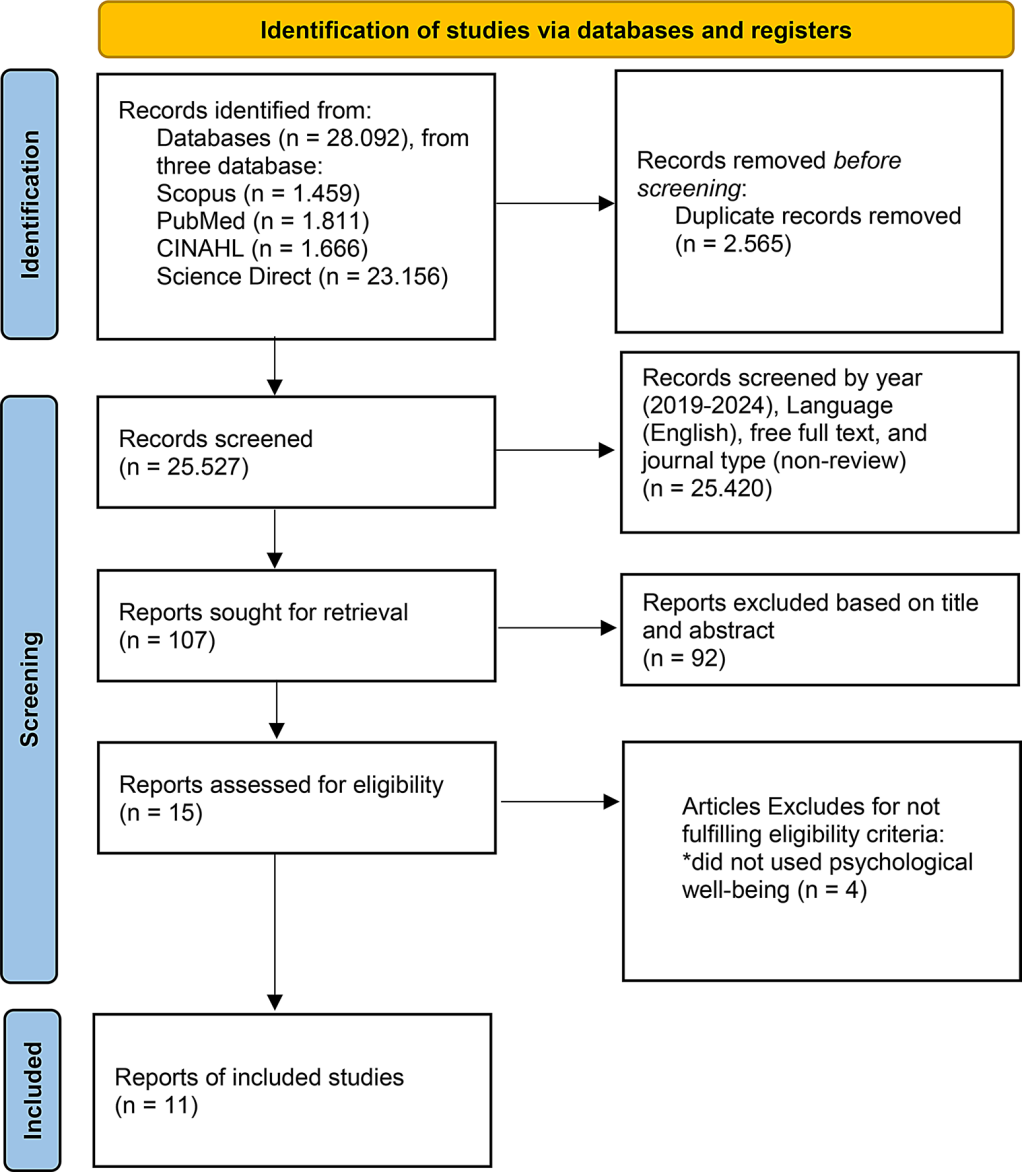
PRISMA flow diagram

Figure 1 illustrates the PRISMA flow diagram used in this scoping review to depict the process of identification, screening, and selection of studies (Fig. 1). Initially, 28,092 articles were retrieved from various databases using predefined search terms. After removing 2565 duplicate results, 24,419 articles were available for the first phase of screening. We excluded based on year (2019–2024), Language (English), free full text, and journal type (non-review), we found 25,420 did not met criteria. After that, we eliminate based on title and abstract, we found that 15 articles met the criteria. Then, we read the full-text of articles, 10 articles met the inclusion criteria and were incorporated into this review.

The scoping review included research articles from diverse geographical locations, studies included in the scoping review were conducted in various countries, including Brazil (3 studies), Turkey (1 study), Norway (1 study), Australia (1 study), Belgium (1 study), Finland (1 study), Canada (1 study), Italy (1 study), Cyprus (1 study), the Netherlands (1 study), Israel (2 studies), Spain (1 study), France (1 study), Denmark (1 study), and Iceland (1 study).The diversity in research settings across different countries underscores the global relevance of addressing bullying and its impact on psychological well-being among adolescents. It also suggests the potential applicability of nursing interventions in various cultural contexts. The research findings suggest that interventions such as the Theater of the Oppressed, the Self-Esteem Development Program, and Solution-Focused Approach peer support groups can be valuable tools in mitigating the negative consequences of bullying and fostering improved psychological well-being. These interventions offer diverse approaches, including creative expression, self-esteem enhancement, and peer support, to address the complex issue of bullying and promote the mental health of young people (Table 1).

**Table 1:** Psychological well-Being intervention of 11 studies articles

No.	Author and Year	Country	Study Design	Population and Sample Characteristics	Intervention	Purpose	Result	Nurse’s Role
1	[12]	Brazil	Quasi-Experimental Study	232 students from the first year of high school aged 10–19 years	Theater of the Oppressed	The aim of this study was to evaluate the impact of an intervention based on Theater of the Oppressed to reduce school bullying among teenagers. This study aimed to assess changes in aggression and victimization before and after the intervention, with a focus on reductions in direct victimization in the intervention group.	Results of intervention use Theater of the Oppressed showed a significant reduction in direct victimization, including physical and verbal aggression, among the intervention group. It states that theater-based interventions, such as those developed in Finland, increase empathy, prosocial behavior, understanding of diversity, and awareness of consequences of bullying, which are aspects related to psychological well-being.	Nurses play a significant role in addressing bullying in schools by promoting good social interactions, self-esteem, and cooperative conflict resolution. They are involved in the prevention and coping with school bullying through dramatization, which includes the use of speech, art, and body movement to engage students at affective and emotional levels. This approach helps in improving empathy, pro-social behavior, and understanding about diversity and the consequences of bullying. The Theater of the Oppressed is highlighted as a strategy that can be incorporated into nursing practice to empower students and improve interactions and quality of life in schools. Nurses are encouraged to engage in intersectoral work and contribute to scientific knowledge production in the field of school violence.
2	[20]	Brazil	Quasi-Experimental Study	The 1,043 teenagers were from grades 5 to 9, aged 10–17 years	Brief anti bullying intervention: Two meetings were held (average duration of 90 min each) with the adolescents in their respective classes and with the teachers	The aim of this research is to evaluate brief anti bullying *intervention* in public schools and analyze its impact on bullying behavior among adolescents. This study aims to assess whether the intervention can significantly reduce bullying behavior over time.	The research results show that brief anti bullying intervention does not have a significant effect in reducing bullying behavior among teenagers. There was no significant difference in bullying scores between the intervention group and the control group after the intervention. Research suggests that the lack of significant results may be due to the brief nature of the intervention and the inclusion of all youth without focusing on specific groups involved in bullying.	The role of nurses in preventing bullying, involves working with teachers in the stages of identification and prevention of bullying and in the implementation of anti-bullying interventions. Nurses can assist and support both teachers and other education and health professionals to identify and intervene in bullying situations. This interdisciplinary and inter-sector action is part of the Health at School Program (PSE), which recognizes the school environment as a privileged place for the development of health actions, including the prevention of bullying.
3	[15]	Australia, Belgium, Finland, Canada, Italy, Cyprus and the Netherlands	Quasi-Experimental Study	39,793 children and adolescents aged 5–20 years	KiVa, NoTrap!, Olweus Bullying Prevention Program, and ViSC.	This study aims to determine the overall effectiveness of this intervention and identify for whom and under what conditions this intervention is most effective. This research also seeks to understand the impact of specific intervention components and highlights the need for specific approaches to effectively address bullying in educational settings.	The results showed that school-based anti-bullying interventions were effective in reducing victimization and bullying among children and adolescents. The intervention demonstrated small but statistically significant reductions in self-reported victimization (d = -0.14) and bullying perpetration (d = -0.07). The intervention was found to be more effective for younger children (under age 12) and for those with higher rates of initial victimization. However, there was no significant variation in effectiveness across other demographic subgroups such as gender, ethnicity, and socioeconomic status. Additionally, certain intervention components, such as non-punitive discipline methods, can have negative effects, particularly among girls and frequent bullies. Overall, while interventions are generally effective, research shows the need for tailored approaches to better address the needs of specific subgroups.	Nurses play a crucial role in supporting the effectiveness of interventions through a holistic health approach. They can contribute to the early detection of the impacts of bullying by screening students’ mental and physical health for signs of anxiety, stress, or psychosomatic disorders. Additionally, nurses can implement empathy-based interventions and social skills training to enhance students’ emotional awareness and resilience against bullying, aligning with findings that interventions targeting cognitive-emotional skills have the potential to reduce the effects of bullying. In implementing school-based interventions, nurses can also collaborate with teachers and other healthcare professionals to ensure that school policy-based programs, such as monitoring high-risk bullying areas and appropriate disciplinary approaches, do not negatively impact vulnerable groups. Thus, the role of nurses is not only limited to clinical aspects but also extends to advocacy and evidence-based policy development to create a safe and supportive school environment for students.
4	[13]	Turkey	Randomized Controlled Trials	The sample size was calculated as a total of 66 students, with 33 students in the intervention group and 33 students in the control group.	Self-Esteem Development Programme	The aim of this research is to test the effect of the Self-Esteem Development Programme on self-esteem and victims of bullying by peers among students. This program aims to reduce bullying by peers by increasing students’ self-esteem and is considered an effective intervention in preventing mental problems caused by low self-esteem and exposure to bullying by peers, thereby potentially increasing psychological well being	The research results show that the Self-Esteem Development Programme significantly reduced self-esteem scores and peer bullying among students. This suggests that the program is an effective psychiatric nursing intervention to increase students’ self-esteem and reduce bullying among peers, which can contribute to improved psychological well-being. Additionally, this study provides significant evidence for increasing self-esteem and reducing peer bullying among students with low self-esteem and at risk for peer bullying, which may benefit their psychological well-being.	Nurses have an important role in identifying bullies and victims, as well as guiding bullying prevention efforts through their responsibilities as educators, counselors, advocates, and agents of change. As essential contributors to school health education, nurse-led programs have been shown to effectively reduce peer bullying and enhance students’ self-esteem. They also help strengthen the confidence of bullying victims and support them in coping with threats. School-based nursing interventions raise awareness about bullying and promote positive behavioral changes among students. In addition, nurses can lead initiatives to educate teachers and families, organize seminars, and improve skills through in-service training.
5	[22]	Norway	Qualitative Study	The study sample consisted of 24 children, aged 11 to 13 years, from one urban school and one suburban school. Four of these children, three girls and one boy, experienced bullying, and 20 participated in support groups.	Solution-Focused Approach (SFA)	The aim of this study was to explore the experiences of bullied children in support groups and how participation in these groups affected the children. This research aims to provide opportunities for change with a Solution-Focused Approach (SFA) and help children to be accepted among peers, thereby increasing their psychological well-being and reducing bullying.	The study found that support groups provide opportunities for change and can help bullied children to be accepted among peers. This involvement and support from peers contributes to psychological well-being of children by providing friendship, strength, and valuable experiences. Both bullied children and those who participated in support groups reported gaining strength and courage through participation, indicating that such interventions can improve psychological well-being and reduce bullying.	The role of school nurses is diverse, encompassing the protection of children’s mental health and the implementation of interventions to prevent bullying in schools. Positioned to lead interdisciplinary collaboration, they emphasize the need for a systemic approach that involves both the school’s social environment and the children’s families. They also offer support and motivation to children in support groups, helping them develop resilience and confidence. Furthermore, school nurses conduct weekly consultations with these groups, encouraging members to propose effective strategies to support bullied or socially excluded children.
6	[21]	Italy, France, Spain, Denmark, Iceland	Randomized Controlled Trials	2845 adolescents and 2430 families in the intervention group, and 1615 adolescents and 2227 families in the control group. Adolescents aged 12–14 years and 51% of them are girls.	UPRIGHT and CREEP Projects	The aim of this intervention-related research is to contribute to current knowledge of evidence-based interventions for prevention (cyber)bullying and improvement psychological well-being. This study aims to assess the effectiveness of resilience training and digital interventions as protective factors to reduce high-risk behavior, such as (cyber)bullying, in school and out-of-school settings. This intervention is designed to engage youth and their communities in the development of life skills, thereby improving mental well-being and achieving educational goals.	This research addresses key challenges and insights gathered during the design and implementation of an intervention, aimed at reducing the risk of (cyber) bullying and improving the psychological well-being of early adolescents. Feasibility and acceptability of these prevention programs is considered key to achieving these outcomes. However, specific results or data on the effectiveness of the intervention in reducing bullying and improving psychological well-being were not detailed in the citations provided.	Nurses play a vital role in preventing bullying and cyberbullying by fostering resilience and well-being among adolescents. They can educate students, parents, and teachers on digital safety, coping strategies, and the psychological impact of cyberbullying. Through collaboration with schools and communities, nurses can help implement evidence-based intervention programs that promote emotional regulation, problem-solving skills, and social competence. They can also support victims through counseling, early detection initiatives, and digital tools to mitigate cyberbullying effects. By integrating mental health promotion into school environments, nurses contribute to reducing bullying-related risks and enhancing adolescent psychological well-being​
7	[24]	Israel	Cross-Sectional Study	2733 children, aged 10–12 years	Developing spiritual-based interventions	Reduction-related goals cyberbullying and improvement psychological well-being involves the development of prevention and intervention programs aimed at addressing bullying among young children. These programs should focus on reducing levels of peer bullying in schools and increasing awareness about the negative consequences of various types of bullying, including cyberbullying, on children’s perceptions of themselves, their life satisfaction, and quality of life.	These findings suggest that higher levels of child religiosity serve as a protective factor that moderates the negative impact of bullying on subjective well-being (SWB) among 10–12 year old children in Israel. Therefore, developing school-based interventions that include religious dimensions, such as faith, trust, and coping strategies, can help strengthen children’s inner resilience and support them in overcoming difficulties related to bullying.	Nurses play a role in directly supporting bullying victims by providing emotional support, educating them on coping strategies, and implementing psychosocial interventions to build resilience. Additionally, they collaborate with schools and communities in anti-bullying programs and offer culturally appropriate spiritual care to enhance victims’ emotional well-being.
8	[40]	Spanish	Randomized Controlled Trial	12 elementary schools and 17 middle schools, ages 8–16	LINKlusive	The aim of this study was to evaluate the efficacy of a web-based intervention in schools called LINKlusive, designed to reduce bullying and improve mental health among children and adolescents aged 8–16 years, particularly those with special educational needs, in Madrid, Spain. This study aimed to assess the effectiveness of the intervention in reducing peer-reported bullying victimization and improving mental health symptoms, self-esteem, and quality of life.	The primary outcome measure of this trial was victimization bullying peer-reported, specifically defined as at least two peer nominations on the Sociescuela victimization subscale. Additionally, the study assessed peer-reported bullying behavior, self-reported victimization and bullying behavior, mental health symptoms (e.g., internalizing and externalizing symptoms, psychotic-like experiences), self-esteem, and quality of life as secondary outcome measures.	Nurses support school-based interventions to reduce bullying and improve the mental health of children and adolescents. They contribute to online education programs for teachers and parents, assist in web-based interventions to enhance students’ empathy and social skills, and collaborate in peer-support strategies to aid bullying victims.
9	[25]	Israel	Cross-Sectional Study	The sample consisted of 507 Israeli high school students who completed all sections of the questionnaire. We excluded 37 participants from the analysis because they did not complete the entire questionnaire. Of the participants included in the sample, 53% were boys and 47% were girls, aged 11–16 years.	Holistic approach and child centered care	The aim to investigate the relationship between being a victim of bullying and adolescents’ sense of well-being, with a focus on resilience and self-concept as mediating factors. This research aims to understand how these mediating factors can provide information for intervention and prevention programs aimed at improving the well-being of adolescents affected by bullying.	The sample consisted of 507 Israeli high school students who completed all sections of the questionnaire. We excluded 37 participants from the analysis because they did not complete the entire questionnaire. Of the participants included in the sample, 53% were boys and 47% were girls, aged 11–16 years,	Nurses play a crucial role in reducing bullying by educating adolescents, parents, and teachers about its impact and coping strategies. They provide psychosocial support to victims, helping them build resilience and self-concept through counseling and peer discussions. Collaborating with schools and communities, nurses can create safe environments and implement prevention programs. They also promote healthy technology use by teaching digital ethics and encouraging victims to report bullying. Additionally, nurses contribute to research and develop intervention programs to enhance adolescent well-being, reinforcing resilience and self-esteem as key protective factors against bullying’s negative effects
10	[23]	Turkey	Cross-Sectional Study	377 students aged 14–19 years	Developing forgiveness skills and adaptive responses	The aim of this study was to investigate the relationship between cyber victimization, coping strategies, forgiveness, and well-being among adolescents. This study aims to confirm that cyberbullying coping mediates the impact of cybervictimization on well-being and to explore the role of forgiveness as a positive predictor of coping behavior. This research seeks to highlight the importance of forgiveness and effective coping strategies in improving the well-being of cyber victims and suggests practical implications for psycho-educational and mental health awareness programs in schools.	The results of the study indicate the following key findings: **Cyber Victimization and Well-Being**: Cyber victimization negatively impacts well-being, confirming that victims experience lower levels of well-being compared to non-victims. **Forgiveness as a Mediator** Forgiveness mediates the relationship between cyber victimization and well-being. Victims who practice forgiveness tend to have better well-being, as forgiveness helps reduce negative feelings and aggressive behaviors. **Coping with Cyberbullying** Coping strategies also play a mediating role in the relationship between cyber victimization and well-being, suggesting that effective coping can enhance well-being. **Model Fit and Analysis** The study employed confirmatory factor analysis and structural equation modeling to validate the scales used and assess the relationships among the variables, finding good model fit indices.	Nurses play a key role in mitigating cyberbullying by promoting coping strategies and emotional resilience among adolescents. They can provide education on digital safety, foster forgiveness as a coping mechanism, and support victims through psychosocial interventions. By collaborating with schools and communities, nurses can implement prevention programs that enhance well-being. Additionally, they can contribute to research on cyber victimization and develop targeted interventions to reduce its psychological impact. Their role in guiding adolescents toward adaptive coping strategies, such as seeking social support and engaging in problem-solving, is crucial in maintaining mental health and emotional stability​
11	[11]	USA	Pre-experimental study	20 adolescents ranged in age from 12 to 16, with an average age of 13.9 years	COPE Intervention (MINDSTRONG to Combat Bullying)	Examined the feasibility, acceptability, and preliminary effects of the MINDSTRONG to Combat Bullying Program for adolescents affected by bullying and mental health symptoms.	The program resulted in significant reductions in adolescents’ depressive, anxiety, and somatic symptoms, as well as bullying victimization frequency, while also improving their personal beliefs.	Nurses serve as facilitators who deliver CBT-based sessions to help adolescents develop coping skills, manage emotions, and build self-confidence. They guide participants through structured psychoeducation and provide support to reduce symptoms of anxiety, depression, and bullying-related distress.

### Education-based approach and school involvement

Educational and school engagement-based approaches focus on fostering collective awareness and enhancing knowledge within the school community to create a safer and more inclusive environment. For example, the Brief Anti-Bullying Intervention by Bottan et al. (2020) [22], which involved two short meetings between students and teachers, initiated discussions about bullying, though it did not significantly reduce bullying behavior. This intervention provided a foundation for more comprehensive initiatives.

The UPRIGHT and CREEP Projects, as described by Gabrielli et al. (2021) [23], offer more systematic approaches: the UPRIGHT Project promotes resilience through school-wide training, while the CREEP Project uses digital tools to detect and prevent cyberbullying. Both emphasize data-supported prevention involving the entire school community.

Programs like KiVa, NoTrap!, Olweus, and ViSC, highlighted by Hensums et al. (2023) [15], demonstrate effectiveness in reducing bullying through collective efforts, though maintaining long-term impact remains a challenge. The LINKlusive intervention, examined by Díaz-Caneja et al. (2021), focuses on inclusivity by providing web-based resources and tailored materials to foster a preventive school culture.

These interventions prioritize creating supportive environments through education, participation, and evidence-based strategies that are tailored to specific contexts such as the targeted student population and the involvement of personnel such as teachers, counselors, and health workers. This contextualized approach allows the intervention to effectively address bullying and promote students’ long-term psychological well-being.

Nurses play a key role as *educators* and *advocates* in education-based approaches. They provide training to teachers, students, and parents on recognizing signs of bullying, prevention strategies, and appropriate interventions. Nurses collaborate with schools to design mental health programs that foster inclusive and safe learning environments. In initiatives like the UPRIGHT and CREEP projects, nurses can serve as facilitators, delivering resilience-building training and assisting in the digital detection and prevention of cyberbullying.

### Psychological approach and personal development

Psychological and self-development approaches to addressing bullying focus on enhancing individuals’ ability to cope with difficult situations by strengthening coping mechanisms, improving self-esteem, and developing psychological skills. These interventions aim to build resilience and improve psychological well-being by targeting emotional and psychological aspects.

The Solution-Focused Approach (SFA) in support groups, as described by Heitmann et al. (2024) [24], empowers bullying victims by involving them in peer-led discussions that focus on practical solutions and future-oriented strategies, providing direct support through individual consultations. Similarly, the Self-Esteem Development Program by Sır and Lok (2024) uses weekly sessions to enhance self-awareness, goal-setting, and self-image, leading to improved self-esteem and reduced bullying [25].

Eroglu et al. (2022) explored the Developing Forgiveness Skills and Adaptive Responses intervention, which uses forgiveness as a coping mechanism to help victims manage negative emotions and recover emotionally, thereby enhancing psychological well-being [26]. These approaches equip individuals with the skills to overcome bullying and maintain long-term psychological health, focusing on empowerment, resilience, and emotional recovery.

The COPE (Creating Opportunities for Personal Empowerment) program is a Cognitive Behavioral Therapy (CBT)-based intervention designed to improve adolescents’ mental well-being, especially in depression [11]. COPE consists of 12 intervention components that have been shown to be effective in various CBT-related studies, with each session including incremental learning and skill practice through homework. Adapting this program for bullying intervention, as in MINDSTRONG to Combat Bullying, adds important elements such as psychoeducation on bullying, emotion regulation training, and social skills reinforcement through simulated bullying situations. The approach also involves parents in two sessions to support their child more effectively. Initial studies show significant results, including decreased depressive, anxiety, and somatic symptoms in adolescent victims of bullying, as well as decreased victimization frequency and increased self-confidence, with strong positive effects.

Nurses act as *counselors* and *therapists* in psychological and personal development approaches. In interventions such as the Solution-Focused Approach (SFA), nurses lead peer discussions that focus on practical, future-oriented solutions for bullying victims. They guide sessions in programs like the Self-Esteem Development Program or COPE, enhancing self-awareness, emotional regulation, and social skills among adolescents. Additionally, nurses educate parents on supporting their children effectively, strengthening the overall impact of these interventions.

### Holistic and cultural approach

Holistic and cultural approaches to bullying interventions go beyond conventional methods by incorporating cultural, spiritual, and artistic dimensions to promote emotional, social, and spiritual well-being. Theater of the Oppressed as studied by Alencastro et al. (2020) [27], uses art and active participation, involving students in role-playing based on their experiences, which significantly reduces both physical and verbal bullying. Spiritual-Based Interventions outlined by Massarwi A et al. (2022) [28], leverages spirituality to help children cope with bullying, showing that higher religiosity improves coping and well-being through internal resources like faith and hope.

The Holistic Approach and Child-Centered Care by Shemesh & Heiman (2021) focuses on understanding each child’s unique needs, recognizing how factors like family, social environment, and individual characteristics shape bullying experiences [29]. This comprehensive approach supports children by enhancing self-esteem and coping skills in a responsive environment. Overall, these holistic and cultural approaches provide a more inclusive perspective on bullying, fostering resilience and empowerment to handle social challenges effectively.

Nurses play a vital role as *holistic caregivers* and *cultural mediators* in holistic and cultural approaches to bullying interventions. They integrate cultural, spiritual, and artistic dimensions into care plans, ensuring that interventions like *Theater of the Oppressed* foster expressive and participatory environments for children to process their experiences and build resilience. In *Spiritual-Based Interventions*, nurses guide children in leveraging faith and internal resources to enhance coping and psychological well-being. Additionally, through *Holistic and Child-Centered Care*, nurses collaborate with families to address individual needs, promote self-esteem, and develop effective coping strategies, creating a responsive and inclusive environment for long-term emotional and social support.

## Discussion

This scoping review aimed to explore nursing interventions designed to address bullying among adolescents and enhance their psychological well-being. The findings highlight that nursing interventions are crucial in addressing bullying by leveraging nurses’ roles as educators, counselors, facilitators, and holistic caregivers. In education-based approaches, nurses collaborate with schools and communities to foster safe environments and promote awareness of bullying prevention strategies. Psychological and self-development interventions showcase nurses’ roles in guiding victims through emotional regulation, self-esteem building, and coping skills enhancement. Additionally, holistic and cultural approaches demonstrate how nurses integrate cultural and spiritual values into care plans, addressing the unique needs of individuals and fostering resilience. These roles underscore the multifaceted contributions of nurses in supporting the psychological well-being of adolescent bullying victims, aligning with the objectives of holistic and evidence-based nursing practices.

The findings of this review highlight that nursing interventions are crucial in addressing bullying among adolescents by leveraging nurses’ roles as educators, counselors, facilitators, and holistic caregivers. In education-based approaches, nurses collaborate with schools and communities to foster safe environments and promote awareness of bullying prevention strategies. Psychological and self-development interventions showcase nurses’ roles in guiding victims through emotional regulation, self-esteem building, and coping skills enhancement. Additionally, holistic and cultural approaches demonstrate how nurses integrate cultural and spiritual values into care plans, addressing the unique needs of individuals and fostering resilience. These roles underscore the multifaceted contributions of nurses in supporting the psychological well-being of adolescent bullying victims, aligning with the objectives of holistic and evidence-based nursing practices.

This scoping review lists several interventions that nurses, in their capacity as educators, advocates, and counselors, can use to collaborate with teachers to prevent bullying and support students’ psychological well-being., including creative approaches like “Theater of the Oppressed,” which empowers victims through creative expression, the Self-Esteem Development Programme that reinforces self-concept, Solution-Focused Approach-based support groups that promote social inclusion, spirituality-based interventions, and the development of forgiveness skills and adaptive responses. These interventions not only focus on reducing bullying behavior but also actively promote problem-solving skills, enhance self-esteem, and facilitate social support, all contributing to improved psychological well-being in adolescents. The findings support the idea that comprehensive and multifaceted interventions are more effective in addressing the impacts of bullying than single approaches, as demonstrated in previous research [30].

Several of these interventions are crucial for enhancing psychological well-being. Previous research has emphasized the importance of interventions aimed at improving social skills among bullying victims [31]^,^ [23]. Programs that enhance social competence can lead to a reduction in bullying incidents, thereby improving participants’ psychological well-being [32]. Collaboration between students, parents, and school support is essential for boosting psychological well-being [33]^,^ [34]. Factors influencing the effectiveness of anti-bullying interventions include both individual and contextual elements. For instance, teacher-related factors such as professional burnout can negatively impact the delivery of anti-bullying programs like KiVa, which are critical for their success [35]. Additionally, interventions based on Acceptance and Commitment Therapy (ACT) and mindfulness are necessary to further enhance psychological well-being [36].

Longitudinal studies show that consistent school involvement can impact the structure of students’ well-being components over time, with positive effects evident in increased life satisfaction and positive emotions [37]. Nurses can collaborate with schools by providing health education and mental health support programs, ensuring that interventions are tailored to address both the physical and psychological needs of students. Additionally, solution-focused intervention programs, when integrated with nursing care, have shown promising results in improving students’ mental well-being by developing their personal resources and resilience [38]. This highlights the importance of a multidisciplinary approach, where nurses actively contribute to creating a supportive environment for students’ holistic well-being.

Psychological and self-development approaches play a crucial role in enhancing individuals’ psychological well-being, particularly in the context of education and professional development. One study indicates that students’ psychological well-being is closely related to regulated self-directed learning processes, where cognitive and emotional aspects are key in shaping life satisfaction and personal growth [39]. Recent research shows that early affiliative memories and experiences of feeling secure in current social relationships positively correlate with girls’ psychological well-being through higher levels of self-compassion and psychological flexibility [40].

A study by Merello (2024) proposes a theoretical framework for talent development, emphasizing that psychological balance cannot be achieved without considering both cognitive and non-cognitive aspects, highlighting the importance of a holistic approach to well-being across various cultural contexts [41]. Namu and Ja (2024) further explore how cultural heritage experiences enhance well-being through a multifaceted approach that includes sensory, emotional, cognitive, behavioral, and relational aspects [42]. Their findings indicate that immersion in culture, rather than merely providing an escape, plays a crucial role in promoting psychological well-being [42].

Nurses play a crucial role in addressing bullying and its negative impact on adolescents’ mental health. As part of the healthcare team, nurses can provide therapeutic interventions such as emotional regulation training, psychoeducation, and social skills enhancement to help adolescents manage stress and build resilience [43]. Additionally, nurses can involve families in the intervention process by offering education and support, enabling parents to create a safe and supportive environment for their children. In community settings, nurses can act as advocates by raising awareness about bullying, promoting a positive school culture, and collaborating with teachers and school authorities to develop effective anti-bullying policies [44]. Through a holistic approach that addresses physical, psychological, and social aspects, nurses can help prevent the long-term effects of bullying on adolescents’ mental health.

The findings of this scoping review underline the critical role of nurses in addressing bullying through tailored interventions that enhance psychological well-being. From a policy perspective, integrating nursing-led mental health programs into school health systems can create a framework for early detection and prevention of bullying-related issues [45]. In practice, nurses can implement evidence-based interventions, such as Solution-Focused Approaches and holistic care models, to provide targeted support for bullying victims. In educational contexts, incorporating bullying prevention and mental health strategies into nursing curricula equips future practitioners with the skills needed to address this pervasive issue. Additionally, the findings highlight gaps in current research, emphasizing the need for longitudinal studies to evaluate the long-term efficacy of nursing interventions [46]. This analysis underscores the importance of a multidisciplinary approach, where nurses collaborate with educators, policymakers, and researchers to build sustainable, bullying-free environments that prioritize the psychological well-being of adolescents.

Nurses play a crucial role in addressing bullying and its negative impact on adolescents’ mental health. As part of the healthcare team, nurses can provide therapeutic interventions such as emotional regulation training, psychoeducation, and social skills enhancement to help adolescents manage stress and build resilience [43]. Additionally, nurses can involve families in the intervention process by offering education and support, enabling parents to create a safe and supportive environment for their children. In community settings, nurses can act as advocates by raising awareness about bullying, promoting a positive school culture, and collaborating with teachers and school authorities to develop effective anti-bullying policies [44]. Through a holistic approach that addresses physical, psychological, and social aspects, nurses can help prevent the long-term effects of bullying on adolescents’ mental health.

The findings of this scoping review underline the critical role of nurses in addressing bullying through tailored interventions that enhance psychological well-being. From a policy perspective, integrating nursing-led mental health programs into school health systems can create a framework for early detection and prevention of bullying-related issues [45]. In practice, nurses can implement evidence-based interventions, such as Solution-Focused Approaches and holistic care models, to provide targeted support for bullying victims. In educational contexts, incorporating bullying prevention and mental health strategies into nursing curricula equips future practitioners with the skills needed to address this pervasive issue. Additionally, the findings highlight gaps in current research, emphasizing the need for longitudinal studies to evaluate the long-term efficacy of nursing interventions [46]. This analysis underscores the importance of a multidisciplinary approach, where nurses collaborate with educators, policymakers, and researchers to build sustainable, bullying-free environments that prioritize the psychological well-being of adolescents.

### Limitation

This review has several limitations. The varied research designs and methodologies among the included studies make it challenging to compare and generalize the results of interventions. Additionally, cultural differences across the analyzed studies—such as variations in societal attitudes toward bullying, family dynamics, and the role of educational systems—may influence the effectiveness and applicability of interventions. For example, interventions emphasizing individualistic approaches may be less effective in collectivist cultures where community involvement is central. Such diversity complicates the identification of universally effective factors in addressing bullying. To strengthen the evidence base, future research should adopt more standardized methodologies, provide clear and detailed descriptions of sample characteristics, and explicitly account for cultural and contextual factors in designing and evaluating interventions.

## Conclusion

The conclusion of this study underscores the critical role of comprehensive nursing interventions in addressing the impact of bullying on adolescents’ psychological well-being. Nurses play a pivotal role as counselors and educators, collaborating closely with parents, teachers, and communities to provide ongoing support and develop tailored interventions. These findings highlight diverse approaches, such as empowerment through creative expression in the “Theater of the Oppressed,” self-esteem development using the “Self-Esteem Development Program,” and solution-focused social support, all of which demonstrate significant potential in improving psychological well-being.

This study emphasizes the necessity for nurses to consider individual and contextual factors, such as cultural differences and social environments, to ensure interventions are tailored to the unique needs of adolescents. Nurses’ involvement in designing and implementing these interventions fosters an environment conducive to optimal psychological recovery. Furthermore, integrating digital technologies into interventions can expand program reach and enhance effectiveness, particularly in the digital era.

## Data Availability

No datasets were generated or analysed during the current study.
